# Ninth International Medical Congress, Washington, D. C., September, 1887

**Published:** 1887-12

**Authors:** 


(TO BE CONTINUED.)

---

### NINTH INTERNATIONAL MEDICAL CONGRESS, WASHINGTON, D. C., SEPTEMBER, 1887.

### SECTION XVIII, DENTAL AND ORAL SURGERY.

REPORTED FOR THE INDEPENDENT PRACTITIONER, BY "MRS. M. W. J."

CONTINUED FROM PAGE 592.

### TUESDAY AFTERNOON SESSION.

Dr. J. E. Cravens, of Indianapolis, read a paper entitled "The Management of Pulpless Teeth."

Dr. Cravens was opposed to the injection of medicines through the root canals, as liable to saturate the dentine and prove injurious to the pericemental membrane. In a pulpless tooth the cementum still maintains a vital connection between the dentine and the pericemental membrane, affording collateral sustenance and preserving the tooth in comparative health, comfort and usefulness for many years; if the pericementum is impaired or its function perverted, then the pulpless tooth becomes a dead body and offensive to the economy. The apical space—a dense, dark crypt—if invaded by a bristle thrust through the nerve canal, admits the light to what then becomes a battle-field, a *champs de mars* for hobby horses with their riders astride. The idea seems to prevail that the root canal was designed solely for the introduction of remedies, but the very nature of the parts suggests that it is impracticable to accommodate much medicine. The pericementum is closely confined between the cementum and the walls of the alveolus, with barely room for the membrane in health. If inflamed it thickens, and needing more space it lifts the root in the socket, the elevated crown becoming painful in occlusion. If medicines are forced in where there is no room, we must have pericemental inflammation, followed by alveolar abscess. Medicines are applied to the walls of the cavity, and not allowed or designed to go beyond; but why ? If the objective point is the congested membrane, the remedies must go through the apical foramen to meet it; but the point of inflammation is sometimes quite remote from the foramen, in molars being usually confined to the angle at the junction of the roots; decay does not locate it. With a large number of practitioners pulp-canals must always be treated before filling. Medicines are injected; the tubuli absorb the fluids, and by capillary attraction they are carried to the periphery of the root; the pericementum is invaded, and the medicines work down along the outside of the roots. In a few weeks—or perhaps years—the effects are seen in an irritating exudation through a fistulous opening, or at the gingival borders, the individual detecting the medicine which was placed in the pulp-canal years previously. Finally the pericementum is hypertrophied, its function as conservator of the cementum is perverted, and the tooth is cast off as a dead body.

The methods of this paper require that the apical end of the canal be closed as soon as free from pus or other fluids and obstructive matter. Clean the canal till laudable. Broken down pulp-tissue should be removed by barbed broaches, gaseous contents by mechanical displacement. Absorbent cotton on a broach, making a swab small enough to pass easily through the canal, avoiding compression, will allow the gases to pass by and out, pure air going in to satisfy the vacuum. This simple process will effectually cleanse a root-canal of sulphuretted hydrogen and other gas. The fluid contents will be absorbed by the cotton of the swab, which should be frequently changed, pressure being carefully avoided that nothing be forced through the apical space. This method recognizes the sanctity which hedges the apical space. Whatever enters there is an impudent intruder, and we get a prompt expression of the indignation of the offended membrane. If pus is formed by any disturbance, it should discharge through the pulp-canal; that is its natural exit. It can be cleaned away by the operator as described, till the canal is laudable, when the apex may be permanently closed. If it forms a fistulous opening, the fistula will accomplish the drainage of pus from the abscess, and it will heal when no more pus is formed, without medication. If the tooth is too sore to allow of opening the pulp-cavity, it is better to wait for a decline of the soreness than to force to a conclusion; in the end this will prove more expeditious. For deciduous teeth the method is much the same, but fill them with phosphate of lime in the magma state, but do not put any medicine in the pulp-canals of deciduous teeth; force is inadmissible, and do not fill close to the end of the root. The roots of deciduous teeth are resorbed after they have been filled with phosphate of lime, and fistulas in the gum surrender in one or two days. While admitting that this system is at right angles to the tenets of the profession, Dr. Cravens hoped it might be given a fair trial, and if found as satisfactory to others as it had been to him, they would gladly "throw physic to the dogs."

DISCUSSION.

This paper raised a storm of severe criticism and disapproval. The discussion was opened by Dr. Thomas Fillebrown, of Portland, Maine, who presented a formal paper. He said that while over-medication was no doubt harmful, it is difficult to say what nature unaided might do. Great and uniform success is claimed by many for non-interference, but there are many different standards of success. In cases of chronic abscess with no fistula, he would not dare to risk closing the apex at once. He would not expect to stop the discharge by closing its outlet, and there would be danger of pyæmia, which he would not have the temerity to risk. He thought when a tooth was "too sore to open," the true remedy was to open at once, saving days of agony by a few moments' pain, and avoiding the process of suppuration by allowing the pent-up contents to escape. The "apisal" space was to him a myth. There was no space between the cortical substance of the alveolar wall and the root of the tooth, unless excavated by art or through absorption by disease. Hypertrophy, discoloration, etc., are more frequently the results of retained pulp than of medication; it is not fair to charge to medication what is due to percolation or osmosis. The treatment of deciduous teeth laid down by Dr. Cravens was not consistent. He says, "use no medicines in deciduous teeth," and then proceeds to fill them with phosphate of lime—an antacid and insoluble. What becomes of it when the roots are resorbed ?

Dr. W. C. Barrett, of Buffalo, N. Y., was astonished at the presentation of such a paper before a world's convention of dentists. He protested against its reception or consideration as an indication of the culture and knowledge and professional training of American dentists. The paper was peculiar in every way. Its etymology was singular and its orthography must be *sui generis,* while its pathology outraged all modern progress. It entirely ignored all the advances of the past twenty-five years. Why any one should go back a generation to revive the long-ago exploded theories of the dark ages, and then parade them before a Congress supposed to be made up of the best minds in dentistry, and to be an exposition of all that is latest and best in professional knowledge, was quite incomprehensible to him. Had the paper been presented before some local society, he should only have entered a mild protest, and taken exception to the "system." But when read before such a meeting as this, he did not feel that such lack of acquaintance with modern practice could be too severely denounced.

In a septic condition of a tooth-pulp, the first thing to be done, under all circumstances, is to give egress to septic matter. The next step is disinfection; this is to be followed by a germicide, and when the whole is rendered aseptic and time has been given for inflammatory products to be removed, the cavity should be most thoroughly sealed against re-infection. That is all there really is to the treatment of septic teeth. The assertion that toxic matter can be neutralized by merely mechanical measures must be made either ironically or without consideration of the subject.

Dr. A. W. Harlan, of Chicago, said that the paper read like a mediæval romance. Repudiating modern advances in antiseptic surgery and bacteriology, the author of the paper banishes mephitic gases by swabbing the canals whence pus is flowing, rendering canals "laudable" in that manner, forgetting that the entire dentine becomes saturated and polluted. The paper is too far behind the age to go forth from this Medical Congress as the opinions of the advanced American dentist of to-day. It is absurd, to say the least, to suggest that we should decry all antiseptic and disinfectant treatment, and go back to the mere mechanical methods of forty years ago. If odors could be mechanically displaced (which we deny), if we stop the drainage from the sac and fill without disinfecting, disaster will surely follow. The paper assumes that it is the practice of the American dentist of to-day to force escharotics into and beyond the apex, when there is no fistula! Is this true? This "system," as a system, is unworthy of our consideration, and unworthy of our knowledge of microbian diseases.

Dr. W. H. Morgan said that the gentleman who had just taken his seat was mistaken in stating that such a "system" had been in vogue within twenty or twenty-five years. If he would go back and read papers published in the journals of 1854-5 he would find that they advocated every therapeutic principle adopted to-day; it was true they did not have many of the remedies used now, but the principles involved were the same, and were as well understood. We have been told not to fill the roots of deciduous teeth, because the roots would be resorbed and the metal left projecting; that is, we would have physiological action in a dead body! A dead tooth may be broken down by chemical action, but not by a physiological process. The only remedy for pulpless deciduous teeth is extraction.

Dr. Butler, of Cleveland, Ohio, could not conceive that any one posted on pathological principles could project such a "system" except with the object of bringing out the opposite side. If this was his object, he had certainly succeeded. He hoped our friends from the other side of the Atlantic would understand this. Certainly the results reached could not have been brought out in any other way, and we should take a charitable view of the paper because of the result.

Dr. Harding, of Shrewsbury, Eng., said that since he had been in America, he had seen much to excite his wonder and admiration, but nothing had astonished him quite as much as the paper under discussion. In England they were imbued with the ideas promulgated by Prof. Lister, based on the researches of Tyndall and others, on the subject of pus and septic matter. To be gravely told that septic products can be gotten rid of by mechanical treatment excites both interest and astonishment. He must think, as one gentleman had suggested, that the paper was written solely with a view to excite discussion, and he was glad to see this expression of general opinion among American dentists. When pus is formed from dead pulp it is certain to infiltrate all the structures around, and as long as germs have pabulum to feed upon they will be reproduced. The opinion that germicides applied to the interior of the canal will produce inflammation of the periosteum is wholly unfounded.

Dr. Cunningham, of England, had hoped to lay before the Section statistics of over five hundred cases filled by the "immediate" method, which might induce them to look more leniently upon the essayist of the afternoon. Unfortunately his papers had not arrived, but he asked permission to lay the matter before them later, as his diagrams and figures might add interest to the discussion.

Dr. Cravens postponed his concluding remarks until after the statistics spoken of had been presented.

Dr. T. E. Weeks, of Minneapolis, read a paper on "Matrices as Adjuncts in Filling Teeth," defining the use of the matrix as the conversion of compound cavities into simple ones having four walls. He spoke of the impossibility of using any one shape or form to the exclusion of all others, and with the aid of a series of charts described a large number of the patents now on the market, showing that, though very numerous, they all depend on the application of a few principles, variously modified by the use of flattened wire, strips and bands, entire circles, partial loops, etc., held in position by wedges, solder, shellac, springs, set screws, bolt and nuts, etc., the essential requisites to constitute the best being close adaptation to the tooth, adaptability to the greatest number of cases, non-corrosive material, and pliability sufficient to admit of flush fillings at the margins of cavities. With all the variety on the market, the dentist still needs the ingenuity to devise and the ability to make, on the spur of the moment, out of material at hand a matrix for the case that nothing ready-made will fit, and not expect to use a matrix in every case, guarding against riding a hobby till it throws him into the ditch.

Dr. S. H. Guilford, of Philadelphia, opened the discussion. He said that the object of the matrix being to enable us to perform a difficult operation with less difficulty, it was unnecessary to argue in favor of its value, though there are men who totally condemn them, their arguments being that the space cannot be absolutely filled, that there is a surplus of material where it is most difficult to draw it off ; that the portion built against a matrix is unlike the natural contour of a tooth, etc. But unless an operator has the skill of a Marshall Webb, which few can hope to attain, with the aid of the matrix both time and material are saved and the work much simplified. The subject was not further discussed.

